# To Diet or Not to Diet This Is the Question in Food-Protein-Induced Allergic Proctocolitis (FPIAP)—A Comprehensive Review of Current Recommendations

**DOI:** 10.3390/nu16050589

**Published:** 2024-02-21

**Authors:** Silvia Salvatore, Alice Folegatti, Cristina Ferrigno, Licia Pensabene, Massimo Agosti, Enza D’Auria

**Affiliations:** 1 Pediatric Department, Hospital “F. Del Ponte”, University of Insubria, 21100 Varese, Italy; afolegatti@studenti.uninsubria.it (A.F.); massimo.agosti@uninsubria.it (M.A.); 2 Department of Pediatrics, Buzzi Children’s Hospital, 20154 Milan, Italy; cristina.ferrigno@unimi.it (C.F.); enza.dauria@asst-fbf-sacco.it (E.D.); 3 Pediatric Unit, Department of Medical and Surgical Sciences, University Magna Graecia of Catanzaro, 88100 Catanzaro, Italy; pensabene@unicz.it

**Keywords:** allergic proctocolitis, FPIAP, diet, cow’s milk, breastfeeding, infants, hydrolyzed formula, rectal bleeding, cow’s milk allergy, CMA

## Abstract

Food-protein-induced allergic proctocolitis (FPIAP) is an increasingly reported transient and benign form of colitis that occurs commonly in the first weeks of life in healthy breastfed or formula-fed infants. Distal colon mucosal inflammation is caused by a non-IgE immune reaction to food allergens, more commonly to cow’s milk protein. Rectal bleeding possibly associated with mucus and loose stools is the clinical hallmark of FPIAP. To date, no specific biomarker is available, and investigations are reserved for severe cases. Disappearance of blood in the stool may occur within days or weeks from starting the maternal or infant elimination diet, and tolerance to the food allergen is typically acquired before one year of life in most patients. In some infants, no relapse of bleeding occurs when the presumed offending food is reassumed after a few weeks of the elimination diet. Many guidelines and expert consensus on cow’s milk allergy have recently been published. However, the role of diet is still debated, and recommendations on the appropriateness and duration of allergen elimination in FPIAP are heterogeneous. This review summarizes and compares the different proposed nutritional management of infants suffering from FPIAP, highlighting the pros and cons according to the most recent literature data.

## 1. Introduction

Food-protein-induced allergic proctocolitis (FPIAP) is a transient, non-IgE-mediated allergic colitis manifesting with rectal bleeding and often associated mucus-streaked stools (with possible diarrhea) in well-appearing and thriving infants, with negative tests for pathogens [1,2,3,4,5]. Disappearance of blood in the stool usually occurs within 72–96 h from eliminating food allergen (most frequently cow’s milk protein, CMP), although it may take weeks and a more restricted diet in some patients. The real prevalence of FPIAP is still uncertain, largely depending on the referred population and diagnostic criteria, ranging from 0.2% [6,7] in healthy infants to 64% of infants with bloody stools [3,8]. Noteworthy, FPIAP often occurs in breastfed infants and may be associated with atopic dermatitis or other minor gastrointestinal symptoms (such as fussing and gagging).

The first case of milk-induced colitis dates back to 1940 [9] and the first case series to 1967 [10]. In 1982, Lake et al. described six breastfed infants presenting bloody diarrhea; fecal leukocytes; focal nodularity with erosions, edema, and increased friability at sigmoidoscopy; inflammatory changes with eosinophilic infiltration at rectal biopsies; and negative infective investigations. All infants responded to CMP-free diet (casein-based hydrolyzed or soy-protein-based formula and partially to a maternal diet) [1].

In the last five years, many clinical studies, position papers, and guidelines from different pediatric scientific societies have been published focusing on diagnostic and therapeutic approaches to infants who suffer from FPIAP. Nonetheless, recommendations are heterogeneous, and the clinical appropriateness, relevance, and duration of exclusion diet in these infants are still a matter of debate [3,5,11,12,13,14].

The aim of this review is to summarize and compare the current recommendations and to highlight the pros and cons of different diet strategies in infants with FPIAP.

## 2. Literature Search

The literature search was based on the MEDLINE database and Google Scholar engine. The following MESH and Boolean terms were used in PubMed: (“Food protein induced allergic proctocolitis” OR “FPIAP” OR “allergic colitis”) AND (“diet” OR “elimination diet”). All types of articles (case reports, clinical trials, reviews, and guidelines) were considered. The search was limited to infants (birth to 23 months) and the English language and was performed from inception to December 2023. The Google database and Google Scholar engine were used to find potentially relevant references in the grey literature. Additional references were retrieved from the included papers.

## 3. Updated Compendium on Pathogenesis of FPIAP

The pathogenesis of FPIAP is still not fully understood, although non-IgE-mediated and cellular immune responses to food proteins involving T cells and eosinophils are generally described [2,3,4,5,11,12,13,14]. Eosinophilic infiltration of the rectal mucosa is reported in many patients submitted to endoscopic investigation, with the number of eosinophils at histology ranging from 6 to more than 20 per 40× high-power field [2]. However, no correlation between the degree of peripheral blood eosinophilia and the rectal eosinophil infiltration has been noted [2], and FPIAP is not associated with other eosinophilic disorders or subsequent development of inflammatory bowel disease. Alteration of specific cytokines has been reported, and they are not mutually dependent on eosinophils. In particular, a decreased expression of transforming growth factor β (TGF-β), produced by regulatory cells, and an increased level of proinflammatory cytokines like tumor necrosis factor-alpha (TNF-α) would alter the epithelial barrier, increase the permeability of tight junctions, and stimulate intestinal inflammation. Nevertheless, a different intestinal microbiota composition (with decreased number of bifidobacteria and lactobacilli) associated with an abnormal innate immune response to food antigens could contribute to impaired oral tolerance to cow’s milk protein or, less frequently, to soy, egg, and corn in infants with FPIAP [3,4,7,15]. Moreover, a genetic predisposition with a positive family history of atopy has been observed in 20–40% of patients [2,3,4,5,15]. In one study, virus particles were detected in the microvillus layer of the colon epithelium in 8 infants with rectal bleeding by electron microscopy but not in the stool samples [15]. The causative role of these findings or the existence of different phenotypes of FPIAP is still uncertain.

## 4. To Diet or Not to Diet

Allergen avoidance is a common recommendation for food allergy management. However, since FPIAP is considered a mild benign transient condition in otherwise healthy infants and spontaneous resolution of bleeding is not infrequent, the diet indication and its beneficial effect are still a matter of debate. Currently, throughout the world, there are heterogeneous recommendations and different clinical approaches [11], as summarized and compared in Table 1.

### 4.1. Rationale for the Pro Diet Approach

Rectal bleeding is an alarming sign for parents and not a physiological finding [12].

FPIAP has been found to be responsible for rectal bleeding in up to 64% of selected cases of infants [8,15]

Endoscopic lesions with focal erythema, erosions, and ulcerations have been found in many patients with rectal and sigmoid biopsies, revealing an inflamed mucosa with eosinophilic and possible neutrophil infiltration that may persist a few weeks or longer [2,15,22].

The timing of mucosal healing has not been adequately assessed as most infants with FPIAP are not submitted to endoscopic investigation. In a (small) prospective cohort study, repeated biopsies were scheduled in infants with persistent rectal bleeding, showing that mucosal inflammation may last up to 5 weeks in selected cases [8]. Lake reported 21 infants who had persistent symptomatic proctitis for more than 6 months [22]. In another cohort study, in 7 (18%) patients, cow’s milk allergy (CMA) persisted at the age of one year, with multiple food allergies detected in 5 out of these infants. In particular, the presence of eczema and inflamed colonic mucosa at presentation were associated with persistence of CMA [15]. Other authors noted that risk factors for the development of multiple food allergies were the presence of atopic dermatitis, high levels of eosinophils at diagnosis, and allergic sensitization (prick test or specific IgE) to food allergens [23,24,25]. Cetinkaja et al. showed that neutropenia (<1500/mm^3^) and eosinophilia (450 mm^3^) were significantly increased in severe cases and that positive IgE for food allergens or non-IgE-mediated multiple food allergies, feeding with cow’s-milk-based formula (at least once during infancy) and, most of all, delayed complementary feeding (OR 5.438 [95% CI, 2.693–10.981], *p* < 0.001) were the predictors for late tolerance [25].

The development of other allergic conditions, late tolerance, or multiple food allergies in non-IgE-mediated cases can be attributed to many different mechanisms involving innate immunity, innate lymphoid and T-regulatory cells, proinflammatory cytokines, eosinophils, abnormal permeability, skin barrier disruption, exposure to allergens, gut microbiota and related metabolites [26,27].

In addition, maternal diet during pregnancy and lactation may also contribute to the duration of FPIAP in infants. According to one recent study, multivitamin supplementation during pregnancy and intake of meat, winter fruits, green vegetables, butter, salt, “ready-to-eat” meals, and pastries during breastfeeding correlated with long-lasting hematochezia [28]. Contrariwise, earlier resolution of FPIAP has been found to be associated with higher maternal education and cheese intake during pregnancy and olive oil consumption during breastfeeding [28].

Mild anemia, despite iron supplements, has been reported in selected patients with persistent bleeding [22] and iron deficiency may then occur in a vulnerable period of life.

Long-term detrimental effects of iron deficiency in early life have been reported [29], but iron status and laboratory results have not been consistently reviewed in patients with FPIAP [6,22].

Moreover, early-life intestinal mucosal inflammation may predispose to functional gastrointestinal disorders (FGIDs) later on in life [30]. In a prospective study of 80 patients with FPIAP, a follow-up protracted to 4 years revealed that significantly more allergic subjects reported FGIDs compared to controls (15% vs. 5%, *p* = 0.035). After adjustment for age and sex, the odd ratio for FGIDs in the FPIAP group was 4.39 (95% CI, 1.03–18.68). FGIDs were significantly associated with iron deficiency anemia, younger age at presentation, and duration of hematochezia, with the last finding confirmed in a multivariate analysis (OR, 3.14; 95% CI, 1.72–5.74) [31]. Intestinal dysbiosis has also been demonstrated in infants with FPIAP [3,15,32], but data on microbiota diversity and dietary intervention effects are still limited. It is interesting to note that the addition of Lactobacillus rhamnosus GG to an extensively hydrolyzed casein-based formula significantly improved rectal bleeding in a group of infants with FPIAP [33].

In the vast majority of patients with FPIAP, hematochezia disappears shortly after eliminating CMP from the maternal and/or infant diet, and multiple food avoidance is rarely necessary.

In the majority of infants cow’s milk tolerance is acquired by 6–12 months of life [15,34] greatly reducing the diet period. By contrast, the presence of blood in the stool is a matter of concern for parents and may cause a reduction or discontinuation of breastfeeding, particularly in mothers who become anxious or feel guilty about their child’s rectal bleeding.

### 4.2. Cons Diet Reasons

The maternal elimination diet is not universally recommended because of mild transient clinical manifestation in FPIAP and possible spontaneous resolution. One prospective study enrolled 40 infants (mean age: 2.7 months) with overt rectal bleeding who were randomized to continue or eliminate CMP for 1 month. Overall, 8 (20%) infants had spontaneous resolution of hematochezia, whereas 32 (80%) infants manifested bloody stools during the follow-up (mean [range]: 2.1 [1,2,3,4,5,6,7,8,9,10,11,12,13,14,15] per day) with a mean number of 6 days of rectal bleeding that was not affected by CMP elimination diet. At colonoscopy, the mucosa appeared normal in less than half of the patients, and histology frequently revealed typical inflammatory findings. Atopic eczema was also present in 38% of the population, positive IgE concentrations or skin-prick tests were uncommon, and all infants showed normal growth parameters [15]. In another study, 11 out of 14 infants with early reintroduction of CMP had no recurrence of rectal bleeding. The authors found that these patients were significantly younger at initial consumption of CMP compared to those who continued the elimination diet (6.7 ± 1.6 months vs. 17.7 ± 9.2 months, *p* = 0.002), with no difference in their hemoglobin levels at 1 year of life [6].

Being on the CMP elimination diet may also have a detrimental impact on the duration of breastfeeding and quality of life [15,35]. In addition, breastfed infants occasionally continue to have intermittent persistent bleeding despite maternal avoidance of food(s) or because of the inability to remove all sources of allergens or difficult compliance to diet [2,22]. Moreover, one study recruiting 95 breastfed infants with FPIAP showed that 33 patients did not respond to CM elimination and needed exclusion of other food allergens (egg, corn, soy, or multiple elimination) with 4 infants who required an amino acid-based formula [22]. In a cohort of 60 infants diagnosed as having FPIAP, all were reported to be triggered by CM, associated with egg in 6.6%, with chicken in 3.3%, with wheat in 1.7%, with potato in 1.7% and with multiple food allergies in 3.3% of cases [36]. In this population, 53% of infants (*n* = 32) acquired tolerance by one year of life, 25% (*n* = 15) by 2 years, 5% (*n* = 3) by 3, and 1.7% (*n* = 1) by 4 years [36].

According to the current guidelines and literature data, the CMP elimination diet in formula-fed infants is commonly based on extensively hydrolyzed casein- or whey-based formula (eHF), with 10% of infants with FPIAP requiring amino-acid-based formula [34,37].

**Figure 1:**
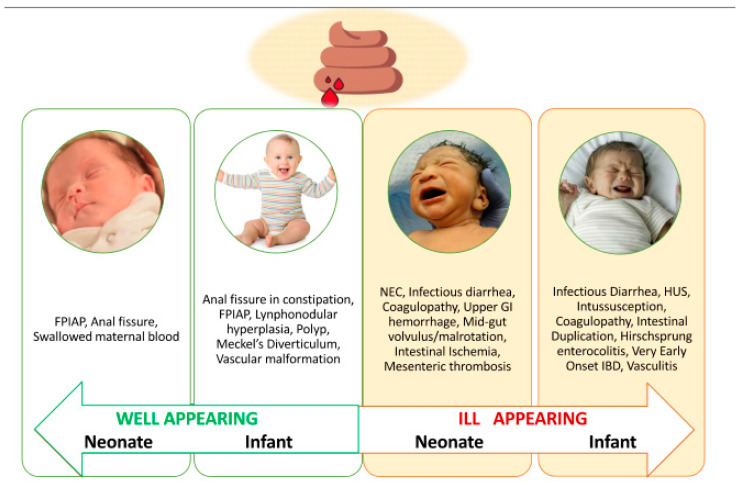
Different causes of hematochezia in neonates and infants according to age and general appearance. Legend: FPIAP = food-protein-induced allergic proctocolitis; NEC = necrotizing enterocolitis, GI = gastrointestinal, HUS = hemolytic uremic syndrome; IBD = inflammatory bowel disease.

In many countries, extensively hydrolyzed or amino acid-based formulas are much more expensive than the standard ones, and reimbursement from the healthcare system presents wide regional differences [13]. In addition, concern arises about taste development, long-term food preference, and possible feeding difficulties for infants who were on the elimination diet [13]. Rectal bleeding is also not a specific sign of allergy in infancy, making misdiagnosis of allergy also possible. Before starting an elimination diet in subjects with bloody stools differential diagnosis, including anal fissures, gastrointestinal infections, Meckel’s diverticulum, infantile polyp, intussusception, volvulus, necrotizing enterocolitis (in neonates), coagulation defects and very early onset of inflammatory bowel diseases (rare) should be considered [3,37]. However, infants with the different conditions above should present other associated symptoms and would not appear healthy (Figure 1).

In 5–42% of breastfed infants, hematochezia may persist during the maternal CMP elimination diet [4]. In these cases, it is recommended to check the mother’s adherence to the diet and, if necessary, eliminate other food protein sources like soy and egg [3,4,14]. However, this extended diet may cause nutritional inadequacy and maternal distress [35].

In one small study including 14 exclusively breastfed infants with long-lasting rectal bleeding not resolving with the oligoantigenic maternal diet, breastfeeding was discontinued, and exclusive feeding with an amino-acid-based formula was started before trying an eHF [38]. However, all guidelines recommend supporting continued breastfeeding in infants with FPIAP, and the use of amino acid-based formulas in these patients has been associated with delayed acquisition of tolerance [7].

### 4.3. The “Watch and Wait” Approach

In 2018 [11], an Italian group first proposed to wait one month from the beginning of rectal bleeding before starting an elimination diet. Moreover, the authors suggested making the challenge soon after the disappearance of hematochezia in infants on diet and continuing the diet for 3 months in case of relapse at reintroduction of the offending food [11]. This early challenge is supported by a study published in 2012 [6], advocated by another Italian group [5] and endorsed by EAACI, WAO DRACMA, and an ESPGHAN position paper [3,12,13]. However, this approach is not generally recommended due to the frequent recurrence of bleeding within 3 days if reintroduction occurs in the first six months of life, as reported by another author [2]. Other studies showed that bloody stools or occult blood disappear in a few weeks, even without an elimination diet [6,15]; thus, a conservative approach of 2–4 weeks may be considered feasible in clinical practice [39].

Based on current literature, the prognosis of FPIAP is good, with possible spontaneous resolution of bleeding in 20% of infants and acquisition of tolerance within 3 and 12 months in most patients (95% according to ASCIA) or rarely up to 3 years of age in selected cases [2,3,8,36,37].

The pros and cons of the elimination diet in infants with FPIAP are summarized in Table 2.

## 5. Other Possible Therapeutic Approach

### 5.1. Probiotics

Microbial diversity with a reduced number of bifidobacteria and lactobacilli has been reported in FPIAP [3,7,15,32]. A specific strain of Lactobacillus rhamnosus (LGG) has shown a beneficial effect on intestinal permeability, mucosal inflammation, and cytokines and on the acquisition of tolerance in a cohort of allergic infants fed an extensively hydrolyzed formula supplemented with this probiotic [40]. In a selected group of infants with FPIAP, the same formula hastened hematochezia resolution and acquisition of tolerance to cow’s milk and significantly decreased the value of fecal calprotectin compared to a control group fed the extensively hydrolyzed formula without the probiotic [33]. Noteworthy, in a double-blind, randomized controlled pilot trial performed in breastfed infants (<6 months of age) with FPIAP, LGG (3 × 10^9^ colony-forming units, twice daily for 4 weeks) (*n* = 14) used as an adjunct to the CM maternal elimination diet did not shorten the duration of hematochezia compared to the placebo group (*n* = 15) [41]. In particular, the mean duration of rectal bleeding was similar in the two groups (17.3 ± 10.6 vs. 15.4 ± 11 days). There was no difference in the number of infants without rectal bleeding within 72 h (2/11 vs. 3/15, relative risk [RR] 0.9, 95% CI 0.2–3.9) or relapse of hematochezia (5/11 vs. 5/15, RR 1.4, 95% CI 0.5–3.5) [41]. On the contrary, 4 cases of FPIAP had resolution of rectal bleeding with LGG monotherapy, without any dietary restrictions. The resolution of hematochezia occurred in 7–28 days [42].

### 5.2. Anti-Inflammatory Drugs

One retrospective study evaluated the effect of mesalamine (5-aminosalicylic acid) treatment in 44/65 infants (mean age 2.98 ± 1.88 months) diagnosed as severe FPIAP due to persistent bleeding (visible blood or mucus in stools in 43/65, 66% of cases and occult blood in the remaining 34% infants) and use of elemental formula [43]. Biopsies showed eosinophilic infiltration in all infants. The intervention group received mesalamine at a dose of 40–60 mg/kg/d dose for an average of 100 days and showed a significantly higher rate of improvement in regurgitation/vomiting, stool consistency, fussiness/irritability, appetite, growth, choking/gagging, back arching, and gassiness compared with the control group. However, no significant difference was observed in dermatitis and blood or mucus in stool. Nonetheless, the group treated with mesalamine showed less hematochezia at reintroduction of cow’s milk (22% vs. 85%) or soy milk (22% vs. 42%) at 15 and 14 months, respectively [43].

According to the benign, mild, and self-limited condition, there is no study using immunosuppressive or biological drugs to treat FPIAP in infants.

## 6. Research Agenda

What emerges from this literature review is that a number of issues still need to be further clarified in infants with FPIAP. First, a better understanding of the exact immune mechanisms involved in this condition will provide pivotal insights for prevention strategy, therapeutic approach, and induction of tolerance. The role of innate immunity, eosinophils, regulatory T cells and cytokines, intestinal microbiota, and viral particles is not fully understood [2,3,14,15,32,44]. Moreover, it is still unclear why the inflammatory response to a food protein is limited to the rectum and sigmoid colon. It has been suggested that the offending protein is bound to antibodies in the breast milk until colonic bacterial enzymes cleave or release the antigen [22]. However, this hypothesis would not explain the presence of FPIAP in exclusive formula-fed infants. The possibility of an autoimmune mechanism has been raised by one study that detected perinuclear antineutrophil cytoplasmic (p-ANCA) antibodies in 24 of 25 infants with FPIAP compared to 0 of 18 controls [45].

Second, the lack of a specific sign and an accurate non-invasive biomarker leads to an overdiagnosis of FPIAP in infants with rectal bleeding who may have a resolution of hematochezia regardless of the elimination diet [46]. Since rectal biopsies and challenge after elimination diet are performed only in selected patients, the exact prevalence of food allergy in infants with rectal bleeding is still uncertain. In one study including 40 infants with rectal bleeding, 20% had spontaneous resolution of hematochezia, while CMA was diagnosed in 7/40 (18%) based on elimination and provocation testing [15]. Third, dietary treatment with allergen avoidance (in most cases, CM elimination diet) is the common intervention in clinical practice. However, in recent years, there has been a growing debate on the real necessity and duration of diet and a heterogeneity in recommendations as reported above. The “watch and wait approach” first proposed in 2018 [11] is increasingly supported by other authors [5], new studies [23], and position papers [3,12,13]. Fourth, the timing of the oral food challenge and reintroduction of the offending food still remains an open question. Some authors suggest making the challenge soon after disappearance of bleeding in infants on diet and then continuing the diet for a limited period of 3 months in case of relapse of hematochezia before rechallenge [3,6,11,17,21], while others recommend the diet for at least 6 months or up to 1 year of life [2,5,12,13,16,18,20]. Fifth, risk factors for persistent allergy, multiple food allergies, and IgE sensitization need to be fully identified [23,24,25,28,36].

As previously mentioned, non-IgE-mediated food allergies often co-occur with IgE-mediated allergies. Buyuktiryaki et al. conducted the first analysis of total IgE and specific IgE levels to common food allergens in a cohort of 257 patients diagnosed with FPIAP, reporting a positive IgE reactive rate of 23% and identifying IgE sensitization as a risk factor for a persistent course in FPIAP [47]. In another cohort, infants with FPIAP showed a double rate of subsequent IgE-mediated food allergy compared to control subjects (11% vs. 5%) [27]. Moreover, a higher prevalence of positive sIgE and/or positive skin-prick test towards allergens has also been found in patients with multiple versus single food allergies [24]. Additionally, the presence of moderate-to-severe atopic dermatitis (AD) has been linked to an increased risk of IgE sensitization [23]. Nevertheless, the role of specific IgE antibodies in predicting FPIAP persistence remains inadequately established; further research is warranted to validate these findings and ascertain the predictive value of IgE antibodies in FPIAP. In conclusion, FPIAP may indeed play a role in the early stages of the non-IgE allergic march but also indicate the possible development of atopic comorbidities.

Lastly, the characteristics of microbiota and the potential use of probiotics in the treatment of FPIAP need further investigation [30,48,49]. 

## 7. Conclusions

FPIAP typically manifests with rectal bleeding in healthy breastfed or formula-fed infants who quickly have disappearance of hematochezia after a food allergen elimination diet. However, spontaneous resolution of bleeding has been reported in some patients, and CMP avoidance is not universally recommended. A “watch and wait” approach (of 2–4 weeks) should be considered, particularly in breastfed infants with mild rectal bleeding. Conversely, the presence of risk factors for persistent bleeding and parental anxiety may favor the choice of an elimination diet. Reintroduction of excluded food after disappearance of hematochezia is recommended both to confirm the diagnosis of FPIAP and to avoid an unneeded prolonged diet. Tolerance to the offending food is acquired in most infants by the age of 1 year but may develop much earlier in some patients and up to 3 years in selected cases. According to recent data, an attempt to reintroduce a normal diet may be considered after 3 months of an elimination diet.

## Figures and Tables

**Table 1:** Summary of the most recent guidelines and position papers on exclusion diet in FPIAP in infants.

Author, Year of Publication	Society/Group of Experts	Recommendations	Duration of Diet	Comments
Miceli Sopo, 2018 [11]	Italian Group	Wait 4 weeks before starting maternal CMP diet or eHF in formula-fed infants with rectal bleeding	Challenge soon after disappearance of bloody stools; continue diet for 3 months if hematochezia reappears	First “Watch and wait” proposal; early challenge
Fox, 2019 [16]	MAP, UK	Elimination trial for 2–4 weeks if CMA suspected, revert to normal diet for 1 week, restart diet if hematochezia relapses	Diet for 6 months or up to 9–12 months of life	Maternal calcium and vitamin D supplementation if breastfeeding
Espin Jaime, 2019 [17]	Spanish Societies	Elimination trial for 1–2 weeks, then challenge and restart diet if hematochezia	Diet for at least 3 months	Assess development of tolerance every 3–6 months
ASCIA, 2019 [18]	Australasian Society (ASCIA)	Elimination trial for 2–4 weeks, then challenge and diet if hematochezia relapses	Diet for 6 months or up to 12 months of life	Maternal calcium supplementation if breastfeeding
Meyer, 2020 [12]	EAACI (on breastfeeding)	A 1-month “Watch and wait” approach in some patients or elimination diet for 2–4 weeks, then reintroduction of allergen	Diet for 6 months or 1 year of age	To start elimination diet, consider also other atopic signs and parents’ distress
Mennini, 2020 [3]	Italian Group	Wait 4 weeks before starting maternal CMP-free diet or eHF in formula-fed infants	72–96 h on diet then challenge; continue diet for 3 months if hematochezia reappears	Exclude soy/egg in maternal diet if no effect of CM-free diet; AAF if no effect of eHF in formula-fed infants
Ebisawa, 2020 [19]	Japanese Society	Elimination diet after challenge if hematochezia. Not specific FPIAP recommendations	Not specified	IgE test for all forms of allergy
Toca, 2022 [20]	Latin American Society SLAGHNP/ LASPGHAN	Elimination diet for 2–4 weeks to continue if hematochezia disappears	At least 6 months or up to 12 months	Maternal calcium and vitamin D supplementation if breastfeeding
Meyer, 2023 [13]	WAO DRACMA	Elimination diet for 2–4 weeks in formula-fed infants, “watch and wait” for one month in breastfed infants	diet for 6 months or up to aged 9–12 months	Dietary advice if prolonged elimination diet
McWilliam, 2023 [21]	WAO DRACMA (on breastfeeding)	A trial of maternal CM avoidance is only advised if the history and examination strongly suggest CMA	Initial diet 2–4 weeks, then attempt to reintroduce CMP	Referral to a dietician is advised. Active support to continue breastfeeding
Vandenplas, 2023 [5]	ESPGHAN	CMP avoidance for severe long-lasting cases. Diagnostic elimination diet for 2–4 weeks	Diet for 6 months or when aged 9–12 months	Diet rarely needed in exclusively breastfed infants

Legend: AAF = amino acid-based formula; CMA = cow’s milk allergy; CMP = cow’s milk protein; eHF = extensively hydrolyzed formulas; FPIAP = food protein induced allergic proctocolitis.

**Table 2:** Elimination diet in infants with FPIAP: pros and cons issues.

Pros	Cons
**Hematochezia Relief:** Eliminating the specific food triggers commonly leads to a reduction or disappearance of rectal bleeding in a few days or weeks	**Nutritional Concerns:** Avoiding certain foods, like cow’s milk, may impact the nutritional balance of the mother’s and infant’s diet.
**Shortened Duration of Hematochezia:** Elimination diet, particularly removing cow’s milk protein, may shorten the duration of FPIAP	**Adherence:** In breastfed infants, poor compliance to the diet is described with occasional recurrent bloody stools due to inadvertent maternal intake of small quantities of offending foods, especially cow’s milk proteins
**Prevention of Complications:** Managing FPIAP through an elimination diet can reduce potential complications, such as mild anemia associated with ongoing persistent bleeding	**Unnecessary Diet:** Up to 20% of breastfed infants with FPIAIP undergo spontaneous resolution of bleeding without changes in the maternal diet
**Improved Quality of Life:** Alleviating distressing signs like rectal bleeding can contribute to an improved family quality of life.	**Distress, Anxiety, and Reduced Quality of Life:** Managing an elimination diet and dealing with the uncertainty of trigger identification can be stressful for parents.
**Reduced Mucosal Inflammation:** Only a minority of infants on diet show persistent colonic inflammation	**Need for Dietician Advice:** In long-lasting and multiple elimination diets, professional dietician advice is recommended to avoid nutritional deficiency and to manage cases with possible unaffordable use of eHF or AAF due to financial constraints
**Possible Reduction of Other Disorders:** Early-life intestinal mucosal inflammation may predispose to functional gastrointestinal disorders	**Risk of Mis-/Overdiagnosis:** Elimination diet without medical supervision and without a close follow-up may delay diagnosis of other diseases or prolong unnecessary diet
**Possible Reduction of Healthcare Costs:** Early disappearance of bleeding on diet may reduce the parental request for medical consultations and investigations.	**Cost**: Extensively hydrolyzed formulas are more expensive than standard formulas and not reimbursed by the healthcare system in many countries. Selected cases may require amino acid-based formulas, which are even more expensive than hydrolyzed formulas.
